# A Pilot Study of Demographic and Dopaminergic Genetic Contributions to Weight Change in Kidney Transplant Recipients

**DOI:** 10.1371/journal.pone.0138885

**Published:** 2015-09-25

**Authors:** Ansley Stanfill, Donna Hathaway, Ann Cashion, Ramin Homayouni, Patricia Cowan, Carol Thompson, Behrouz Madahian, Yvette Conley

**Affiliations:** 1 Health Promotion and Development, University of Pittsburgh, Pittsburgh, Pennsylvania, United States of America; 2 College of Nursing, University of Tennessee Health Science Center, Memphis, Tennessee, United States of America; 3 National Institute of Nursing Research, National Insitutes of Health, Bethesda, Maryland, United States of America; 4 Bioinformatics, University of Memphis, Memphis, Tennessee, United States of America; 5 College of Nursing, University of Kentucky, Lexington, Kentucky, United States of America; University of Maryland School of Medicine, UNITED STATES

## Abstract

Kidney transplant recipients often experience a significant amount of weight gain in the first year post-transplantation. While demographic factors such as age, race, and sex have been associated with weight gain in this population, these factors do not explain all of the variability seen. A number of studies have suggested that genetics also plays a critical role in weight changes. Recently, alterations in the activity of the neurotransmitter dopamine have been associated with weight change, and gene expression studies in kidney transplant recipients have supported this association. The purpose of this pilot study is to first examine the feasibility and methodology, and then to examine the associations of age, race, sex, and genotype for 13 SNPs and 3 VNTRs in 9 dopaminergic pathway genes (*ANKK1*, *DRD2*, *DRD3*, *DRD4*, *SLC6A3/DAT1*, *MAOA*, *MAOB*, *COMT*, *CPE*) for associations with percent weight change at 12 months post-transplantation. Seventy kidney transplant recipients had demographic and clinical data collected as a part of a larger observational study. DNA was extracted from repository buffy coat samples taken at the time of transplant, and genotyped using Taqman and PCR based methods. Three SNPs were independently associated with percent weight change: *ANKK1* rs1800497 (r = -0.28, *p* = 0.05), *SLC6A3*/*DAT1* rs6347 (*p* = 0.046), and *CPE* rs1946816 (*p* = 0.028). Stepwise regression modelling confirmed the combined associations of age (p = 0.0021), *DRD4* VNTR 4/5 genotype (*p* = 0.0074), and *SLC6A3*/*DAT1* rs6347 CC genotype (*p* = 0.0009) and TT genotype (*p* = 0.0004) with percent weight change in a smaller sample (n = 35) of these kidney transplant recipients that had complete genotyping. These associations indicate that there may be a genetic, and an age component to weight changes post transplantation.

## Introduction

Approximately one third of all kidney transplant recipients will gain a significant amount of weight (6–13 kilograms) during the first year after transplantation [1, 2]. This excessive weight gain negatively affects cardiovascular health and contributes to an increased incidence of diabetes [3]. Causes traditionally proposed for the weight gain following kidney transplantation include an improved appetite due to the resolution of uremia and side effects of standard anti-rejection medications [4, 5]. However, research has shown steroid based immunosuppressive protocols do not have a clinically significant effect on the amount of weight gained [4, 6–8]. Additionally, it has been found that there is little difference in physical activity and nutritional intake between kidney transplant recipients who do and do not gain weight [4, 9].

A small amount of the variance in weight change can be explained by certain demographic characteristics (age, race, and sex), with older African American women being the most likely to gain weight [1]. However, these demographic factors alone cannot predict who will and who will not gain weight. The variability in weight change as well as failure to find lifestyle or treatment related factors associated with post-transplant weight gain suggests that genetic factors may have a role in the differential weight change experienced by kidney transplant recipients.

Dopamine is a neurotransmitter that has previously been implicated in substance addiction [10, 11], but dopamine may also play a role in models of food addiction behaviors. For example, dopamine receptor genes and genes related to overall dopaminergic activity have been associated with obesity, increased weight, and food addiction [12, 13]. Gene expression studies conducted in a subset of these same kidney transplant recipients confirm that expression of some dopaminergic pathway genes in adipose tissue negatively correlates with weight change [14]. The genes differentially expressed included the dopamine receptor genes 2, 3, and 4 (*DRD2*, *DRD3*, and *DRD4*), the dopamine transporter gene (*SLC6A3*/*DAT1*), the dopamine degradation enzymes monoamine oxidase A and B (*MAOA*, *MAOB*), and catechol-O-methyltransferase (*COMT*) [14]. The enzyme carboxypeptidase E gene (*CPE*) was also differentially expressed [14]. Although *CPE* is not typically placed within the dopaminergic pathway, there is some evidence that it could affect the dopamine transporter’s efficiency [15].

All of the genes in the dopaminergic pathway have polymorphisms (such as single nucleotide polymorphisms (SNPs) or variable number tandem repeats (VNTRs)) that could cause gene activity to be altered and subsequently place individuals at a greater risk for gaining weight. Identification of individuals as being at a genetically or demographically higher risk for weight gain would enable implementation of personalized lifestyle interventions aimed at reducing environmental contributions to weight gain. The aims of this study are to 1) evaluate the ability of selected dopaminergic polymorphisms to predict percent weight change in kidney transplant recipients and 2) determine the relationship of polymorphisms predictive of percent weight change with demographic characteristics (age, race, and sex) and their combined ability to predict percent weight change in a kidney transplant population. This pilot study will examine the feasibility and methodology of this technique in order to replicate the results in a larger cohort in the future.

## Methods

### Study design and setting

A candidate gene association approach was used to study percent weight change in a subset of kidney transplant recipients with repository samples obtained from participants in a larger prospective study. Subjects were voluntarily recruited and consented from all patients at a midsouth regional transplant center awaiting kidney transplantation and meeting inclusion/exclusion criteria from August of 2007 until March of 2011.

### Sample

This study represents repository subjects from a larger observational study on demographic and genetic factors related to weight after kidney transplantation. The larger study included men and women between 18 and 70 years old, receiving their first transplant, and who were able to read and write in English. Exclusion criteria for the larger study were multiple organ transplantation, previous kidney transplantation, or a nonfunctioning graft during any point in the study period. Participants in the larger study had age, race, and sex information collected, as well as marital status information. Baseline weight and height and 12 month post-transplant weight were also collected. Height data allowed the calculation of body mass index (BMI), as well as changes in BMI at one year post-transplantation (kilograms per meter squared (kg/m^2^). Percent weight change at 12 months was calculated as a percentage of total pretransplant body weight. These variables were shared in a deidentified format. Additionally, participants in the larger study had blood taken at the time of kidney transplant surgery. Buffy coat samples were frozen and stored in a -80°C freezer. The participants in this study also had to sign a repository consent form to allow their biospecimens and genetic data to be used in future work. This protocol was approved by the Institutional Review Board.

### Laboratory Analyses

DNA was extracted from the buffy coat white blood cells using the Qiagen Flexigene DNA kit. For SNPs, genotyping was performed using commercially available TaqMan® assay kits specific for each polymorphism (rs4680, rs4818, rs6277, rs6280, rs12364283, rs1800497, rs6350, rs6347, rs1583645, rs1946816, rs34516004, rs1799836). All TaqMan® assays were conducted on the ABI Prism 7000 Sequence Detection System. VNTRs were genotyped using polymerase chain reaction (PCR) based methods and gel electrophoresis (*MAOA*, *SLC6A3*/*DAT1*, *DRD4*) (S1 Table). The resultant bands were compared to a genetic ladder and to control samples of known, sequenced, genotype. For quality control, all genotypes were determined by two individuals, both blinded to the outcome of percent weight change.

### Statistical Analysis

Demographic variables were collected from clinic records. Age was recorded in years and fractional months at time of transplantation. The outcome of percent weight change was calculated by the formula ((12 month weight-baseline weight)/baseline weight)*100). Descriptive data were presented as means, standard deviations, and ranges. The same data were also examined with appropriate correlations, t tests, chi-squares, and ANOVA statistical tests using R statistical software package, version 2.14.1 [16].

To evaluate the ability of dopaminergic polymorphisms to predict percent weight change in kidney transplant recipients (aim 1), each polymorphism was analyzed using ANOVA, chi squares, and point biserial correlations as appropriate. All analyses were conducted initially using a dose dependent approach. In a dose dependent approach, the assumption is made that a genotype AA is different from Aa, and each of those genotypes is different from aa when considering the outcome of percent weight change post kidney transplant. Therefore, 3 groups (homozygous for risk allele, heterozygous, and homozygous for the nonrisk alleles) are used to compare the impact of genotype on percent weight change.

Next, analyses were conducted in a risk allele fashion. In this type of analysis, those individuals either homozygous (AA) or heterozygous (Aa) for the risk allele for increased percent weight change post kidney transplant (A) are analyzed together as one group and compared to those not carrying a risk allele (aa, and another alternate allele genotypes as appropriate). Genetic variants identified as approaching significance (using the standard p<0.20 [17]) were characterized as having the potential to predict weight change, and were included with demographic characteristics in subsequent regression modelling for aim 2.

In regression modelling, the variables were first assessed for possible multicollinearity. Any variables that were found to be highly correlated (by variation inflation factor (vif)>10) were removed from the model [18]. Then a forward stepwise regression model was built with the remaining variables. These analyses were performed using the R statistical software, package “car”, version 2.14.1 [16].

## Results

### Demographics and Weight Change

Demographic characteristics for the 70 participants in this study are listed in Table 1. The sample was predominantly middle aged, married, African American men. A weak negative correlation was found between age and percent weight change at twelve months’ post-transplantation (r = -0.32, *p* = 0.006). A one tailed t-test found no difference in percent weight change by sex (*p* = 0.23). Likewise, no difference was found for percent weight change by race (*p* = 0.77).

**Table 1:** Demographics of the study sample.

**Characteristic**	**n**		**%**
Sex			
Male	40		57
Female	30		43
Race			
Caucasian	26		37
African American	40		57
Multiracial	2		3
American Indian	2		3
Marital status			
Married	35		50
Single	11		16
Divorced/separated/widowed	8		11
Did not answer	16		23
	**Mean**	**SD**	**Range**
Age (years)	50.7	13.2	19.0–74.0
Baseline weight (kilograms)	82.7	17.9	48.7–122.3
12 month weight (kilograms)	83.9	21.1	44.0–133.9
Percent weight change	1.4	11.6	-22.2–33.5

### Comparison of Genotype in Dose Dependent Manner

The first analysis of genotype data was conducted in a dose dependent manner, which assumes that the homozygous risk allele genotype is different from either the heterozygous or the homozygous non-risk allele genotype. Thus, if found to be significant this means that having the risk allele for increased percent weight change in either one or two doses makes a difference in the overall outcome of percent weight change (Table 2). Percent weight change correlated with *ANKK1* rs1800497 (r = -0.28, *p* = 0.05), and *MAOB* rs1799836 SNPs (r = 0.12, *p* = 0.16). Additionally, *SLC6A3*/*DAT1* rs6347 and *CPE* rs1946816 were significantly (*p* = 0.04 and 0.02, ANOVA) associated with percent weight change. Lastly, *SLC6A3/DAT1* VNTR met criteria for inclusion in a regression analysis (*p* = 0.18, chi-square), as did *ANKK1* rs1800497 (*p* = 0.18, ANOVA).

**Table 2:** Dose dependent comparison of genotype with percent weight change.

					Point biserial correlation coefficient	
Gene	Variant	Risk allele	ANOVA p value	Chi-squared p value	r	p value
*ANKK1*	rs1800497	T	0.18	0.92	-0.28	0.05
*DRD2*	rs6277	T	0.96	0.99	NA	NA
*DRD2*	rs12364283	C	0.91	0.74	-0.03	0.41
*DRD3*	rs6280	C	0.99	0.96	NA	NA
*DRD4*	VNTR	2	0.91	0.97	NA	NA
*SLC6A3/DAT1*	VNTR	10	0.56	0.18	NA	NA
*SLC6A3/DAT1*	rs6347	A	0.04	0.78	0.16	0.17
*SLC6A3/DAT1*	rs6350	A	0.43	0.64	NA	NA
*COMT*	rs4680	G	0.92	0.99	-0.06	0.30
*COMT*	rs4818	G	0.82	0.98	-0.04	0.41
*MAOA*	VNTR	4	0.72	0.70	NA	NA
*MAOB*	rs1799836	A	0.49	0.82	0.12	0.16
*CPE*	rs1583645	A	0.46	0.88	NA	NA
*CPE*	rs1946816	C	0.02	0.28	0.11	0.27
*CPE*	rs34516004	C	0.38	0.58	NA	NA

### Comparison of Genotype by Risk Allele

Risk allele analyses increases power by collapsing categories based upon the presence of the risk allele, regardless of whether it is present in either one or two doses. For instance, if the risk allele is A, then heterozygous and homozygous individuals carrying the A allele are treated as one group and compared to those individuals homozygous for the other alleles. Risk allele chi-squares showed a significant effect for the A risk allele *SLC6A3/DAT1* rs6347 (*p* = 0.0003), and for the C risk allele of *CPE* rs1946816 (*p* = 0.00004) (Table 3). The C allele of *CPE* rs1946816 also showed a significant negative correlation with percent weight change (r = -0.22, *p* = 0.05). Several other variants approached a level of significance that warrants inclusion in a regression model (*p*≤0.20, i.e. the *SLC6A3/DAT1* VNTR, the Taq1A SNP, and the *DRD4* VNTR).

**Table 3:** Comparison of genotype by risk allele.

					Point biserial correlation coefficient	
Gene	Variant	Risk allele	One tail t test p value	Chi-squared p value	r	p value
*ANKK1*	rs1800497	T	0.36	0.99	0.04	0.36
*DRD2*	rs6277	T	0.39	0.99	-0.04	0.39
*DRD2*	rs12364283	C	0.29	0.98	-0.05	0.33
*DRD3*	rs6280	C	0.50	0.96	0	0.50
*DRD4*	VNTR	2	0.17	0.85	0.13	0.15
*SLC6A3/DAT1*	VNTR	10	0.19	0.99	0.1	0.20
*SLC6A3/DAT1*	rs6347	A	0.92	0.0003	0.01	0.46
*SLC6A3/DAT1*	rs6350	A	NA	NA	NA	NA
*COMT*	rs4680	G	0.40	0.99	-0.03	0.40
*COMT*	rs4818	G	0.28	0.99	-0.07	0.28
*MAOA*	VNTR	4	0.33	0.96	-0.05	0.33
*MAOB*	rs1799836	A	0.25	0.99	-0.08	0.25
*CPE*	rs1583645	A	NA	NA	NA	NA
*CPE*	rs1946816	C	0.10	0.00004	-0.22	0.05
*CPE*	rs34516004	C	NA	NA	NA	NA

### Regression Modeling

Only polymorphisms with a p≤0.20 in previous statistical analyses were included in the regression model [17]. Based upon this criterion, the following were included in the regression modelling: rs1800497, *DRD4* VNTR, *SLC6A3/DAT1* VNTR, rs1799836, rs6347, and rs1946816. These variables, along with the demographic characteristics age, race, and sex, were first assessed for possible multicollinerarity using the criterion of vif>10 [18]. This analysis removed the genetic variables rs1800497 (AG genotype), *DRD4* VNTR (4/4 genotype), and *SLC6A3/DAT1* VNTR (8/10, 10/10, and 11/11 genotypes).

Then the remaining variables were included in a forward stepwise regression model (age, race, sex, rs1800497 (GG genotype), *DRD4* VNTR (2/4, 3/4, 4/5, and 5/5 genotypes) *SLC6A3/DAT1* VNTR (9/10 and 10/11 genotypes), rs1799836, rs6347, and rs1946816). For this model, sample size was reduced to 35 from the total N of 70 subjects due to incomplete genotyping for some variants. The model was significant (*p* = 0.0001), with the variables age (*p* = 0.0021), *DAT1* 9/10 genotype (*p* = 0.0199), *DRD4* 4/5 genotype (*p* = 0.0074), rs6347 CC genotype (*p* = 0.0009), and rs6347 TT genotype (*p* = 0.0004) all being important contributors to percent weight change (Table 4).

**Table 4:** Stepwise regression analysis results (n = 33).

Coefficient	Estimate	SE	T value	Pr(>|t|)
Intercept	35.6739	7.7459	4.606	0.00005
Age	-0.4627	0.1387	-3.336	0.0021
DAT1-9/10	10.0966	4.1314	2.444	0.0199
DRD4-4/5	-27.823	9.7744	-2.847	0.0074
rs6347.CC	-15.1582	4.1993	-3.61	0.0009
rs6347.TT	-14.3434	3.6229	-3.959	0.0004

Residual standard error: 8.936 on 34 degrees of freedom, multiple R-squared: 0.51, adjusted R-squared: 0.43, F-statistic: 6.98 on 5 and 34 DF, p-value: 0.0001

## Discussion

This pilot study was conducted to evaluate the feasibility and methodology to uncover the associations of dopaminergic polymorphisms and demographic characteristics with 12 month percent weight change in kidney transplant recipients. Kidney transplant recipients are an ideal model population for studies of weight change as they are likely to gain a substantial amount of weight in the first year after transplantation. However, since not all kidney transplant recipients gain weight, they provide the desired phenotypic (or the observed phenomenon controlled by genetic alterations, here the phenotype was percent weight change) variability for genetic and demographic association studies. In our sample, 25 people gained greater than 5% of their pretransplant body weight, 20 people stayed within ±5% of their pretransplant body weight, and 25 people lost greater than 5% of their pretransplant body weight. This supports previously findings that a minimum of 1/3 of all kidney transplant recipients gain a significant amount of weight within the first year following transplantation [1, 19, 20].

It is important to note that an increase in percent weight change does not necessarily signify that an individual became overweight or obese by BMI classification. Many individuals stayed in the same weight classification, while others lost weight. Only four individuals gained enough weight to become newly classified as obese. Unless the subject was severely underweight at the time of transplant, any increase in percent weight could potentially become problematic and over time lead to obesity. No individual included in this study was underweight at the time of transplant. One subject was categorized as normal weight at the time of transplant, but lost weight and then became classified as underweight at 12 months post-transplant (baseline BMI = 19.91, 12 month BMI = 17.15).

The demographic characteristics evaluated in this study were age, race, and sex. In our sample, race and sex were not found to be associated with percent weight change at 12 months. In earlier work, it has been suggested that older African American women were the most likely to gain weight after transplantation [1, 19]. But in our sample we only found age to be a contributing factor, with less weight change associated with increasing age.

Three of the SNPs (rs1800497, rs6347, and rs1946816) tested in this study were independently associated with percent weight change at twelve months post-transplantation. The first is a SNP rs1800497 (r = -0.28, *p* = 0.05). This SNP lies in the ANKK1 gene, however it is known to impact density of the dopamine receptor, type 2. The association found in this study occurred in a dose dependent manner, indicating that the genotype TT is different than either the TC or CC genotype in the effect on percent weight change of kidney transplant recipients at 12 months. This finding is consistent with previous findings that the C allele is associated with higher numbers of D2 receptors in the brain [21], and also with increased BMI [22] [23].

Importantly, rs1800497 influences the promoter region for the *DRD2* gene. As such, changes in this control region may result in large changes in the amounts of product produced by that gene. The fact that this SNP was found to be associated with percent weight change in this study is consistent with the results of previous work in the transplant population. Changes in expression levels of this gene in adipose tissue of 26 of these same kidney transplant recipients were significantly associated with weight change at six months post-transplant [14].

The second SNP independently associated with percent weight change was the *SLC6A3/DAT1* rs6347. The protein product of this gene is the dopamine transporter, which is responsible for removing dopamine from synapses in the brain. Although the amino acid sequence of the resultant protein is not changed between the two possible alleles (G or A, serine/serine), this SNP showed a dose dependent effect in this sample, and also an effect for the risk allele A. The results found in this study agree with previous associations between the A allele and overweight/obesity [24]. It is possible that this SNP is in linkage disequilibrium (tends to be inherited together) with another causative SNP, or there may be some unknown difference in gene expression between the two possible alleles, despite no overall change in the final protein amino acid sequence.

The last SNP independently associated with percent weight change at twelve months was rs1946816 in *CPE*. While *CPE* has not commonly been considered a dopaminergic pathway gene, there is growing evidence that it could interact to change the rate of dopamine reuptake via the dopamine transporter [15]. This rs1946816 SNP showed dose dependent and risk allele effects. This SNP is located in an intron. Even though the change is not included in a final protein transcript, it may be a serve as a marker for another causative polymorphism. Furthermore, it could influence the likelihood of expression. It is important to note that the expression of *CPE* in adipose tissue of kidney transplant recipients was found to be significantly associated with weight change [14].

While these three SNPs hold promise for future work, these independent associations in such a small sample could be the result of population stratification. This sample was heterogeneous in racial background, making it likely there is a representation of several different ancestral lines containing different minor allele frequencies. For example, the *DRD2* SNP rs6277 has a minor C allele frequency of 0.396 in Caucasian populations. This means that 39.6% of the alleles carried by Caucasian individuals are minor C alleles. In contrast, African American populations have a minor allele frequency of 0.913 (HapMap, February 2014, GRCh38).

Another explanation for potential population stratification could lie in the characteristics of individuals who may be more prone to become a candidate for kidney transplantation. To the best of our knowledge, there is no known relationship between dopamine and kidney transplantation. However, it is possible that some inherent dopaminergic related characteristics are present in individuals with kidney disease and these increase the likelihood that they will require a transplant. Perhaps a reduction in dopaminergic reward from certain behaviors could be related to a reduction in health promoting behaviors. Over time, this could lead to an increased likelihood of kidney failure and the need for transplantation, thereby resulting in an over-selection for this genotype when sampling kidney transplant recipients.

For exploratory purposes, a forward stepwise regression model was built including all demographic characteristics (age, race, sex) and the seven polymorphisms meeting the p≤0.20 criterion (rs1800497, *SLC6A3/DAT1* VNTR, *DRD4* VNTR, rs1799836, rs6347, and rs1946816). Although sample size was greatly reduced (n = 35) and thus the model is underpowered, the model was predictive of percent weight change (*p* = 0.0001, multiple R squared = 0.51, Table 4). This significant result speaks to the contributions of age and genetic characteristics to the outcome of percent weight change for kidney transplant recipients one year after surgery. The model confirmed the significant results found for age, *DRD4* genotype, and rs6347 genotype.

Even though these results are intriguing, there still remains a large percentage of unaccounted variance in post-transplant weight changes. This is somewhat expected for a complex process such as weight gain. It is possible that although the polymorphisms built into the model are important in determining post-transplant weight change, the ones measured for this study do not capture all of the relevant biological pathways for this outcome. This possibility paves the way for future work in discovering what other polymorphisms may predispose kidney transplant recipients to gain weight in the first year after surgery.

### Limitations

This study was conducted as a pilot study to test the methodology and the feasibility in extending the study to a larger sample. As such, the study was underpowered, primarily due to a limited sample size. Generalizability was reduced by the demographic characteristics of the sample being representative of the Caucasian and African American populations, but not of kidney transplant recipients of other racial and ethnic backgrounds.

Additionally, outcome variables other than percent weight change (such as baseline and 12 month blood chemistry panels, cholesterol, and blood pressure measurements) were not considered in this pilot work. Many of these values could hint to future comorbidities for those individuals gaining excessive weight in the first 12 months post transplantation. These values should be collected and examined along with demographic and genotype information in future work. Exclusion criteria related to edema should also be added in these future studies.

A further limitation for this study is that within gene variation was not completely sampled. While many of the most widely studied polymorphisms were tested, this study did not include an exhaustive list. Additionally, there could be other regions of variation within these genes that have not yet been identified, and hence were not sampled in this study. The genotype of the donor kidney was also not considered for this study, which has been shown to impact blood pressure in animal models [25].

### Conclusions

This pilot study was conducted to assess the value of dopaminergic polymorphisms and demographic characteristics (age, race, and sex) for predicting percent weight change at 12 months’ post kidney transplantation. Although the results are somewhat tempered by the small sample size that was used for this study, age at time of transplantation was an important factor in determining the amount of weight change at one year. Genotype for the *DRD4* VNTR, and the *SLC6A3*/*DAT1* SNP rs6347 also have predictive value for percent weight change. Replications of this study with a larger sample size should be done to fully assess the contributions of these polymorphisms to prediction of post transplant weight gain. Expansions and replications of this study may help to develop future genetic testing to better identify kidney transplant recipients most at risk for weight gain. In the meantime, medical professionals should continue to educate all kidney transplant recipients, but most especially younger patients, about the possibility of substantial weight gain in the first year post-transplantation. These patients should be directed to individualized intervention therapies aimed at reducing the amount of weight gained after surgery.

## Supporting Information

S1 Table VNTR genotyping. VNTRs were genotyped using polymerase chain reaction (PCR) based methods and gel electrophoresis (*MAOA*, *SLC6A3*/*DAT1*, *DRD4*).
(DOCX)
