# Supplementary material for: A Pilot Study of Demographic and Dopaminergic Genetic Contributions to Weight Change in Kidney Transplant Recipients
Source: PLoS One. 2015 Sep 25;10(9):e0138885. doi: 10.1371/journal.pone.0138885 (PMC4583246; doi:10.1371/journal.pone.0138885)
Supplement: S1 Table — VNTRs were genotyped using polymerase chain reaction (PCR) based methods and gel electrophoresis (MAOA, SLC6A3/DAT1, DRD4). (DOCX) [file pone.0138885.s001.docx]

**Table 1. VNTR genotyping methods.**

| **Gene** | **Primer set** | **Temperature conditions** |
| --- | --- | --- |
| *MAOA* | **F:** ACA GCC TGA CCG TGG AGA AG | Step 1: 95° for 1 minute |
|  | **R:** GAA CGG ACG CTC CAT TCG GA | Step 2: 55° for 30 seconds |
|  |  | Step 3: 72° for 1 minute |
|  |  | Step 4: Repeat step 1 35 times |
|  |  | Step 5: 10° forever |
|  |  | Step 6: end |
| *DRD4* | **F:** CTT CCT ACC CTG CCC GCT CAT GCT GCT GCT CTA CTG G | Step 1: 95° for 30 seconds |
|  | **R:** ACC ACC ACC GGC AGG ACC CTC ATG GCC TTG CGC TC | Step 2: 70° for 36 seconds |
|  |  | Step 3: 72° for 40 seconds |
|  |  | Step 4: Repeat step 1 40 times |
|  |  | Step 5: 72° for 10 minutes |
|  |  | Step 6: 10° forever |
|  |  | Step 7: end |
| *SLC6A3/DAT1* | **F:** TGT GGT GTA GGG AAC GGC CTG AG | Step 1: 95°C for 30 seconds. |
|  | **R:** CTT CCT GGA GGT CAC GGC TCA AGC | Step 2: 57°C for 36 seconds. |
|  |  | Step 3: 72°C for 40 seconds. |
|  |  | Step 4: Repeat step 1 35 times. |
|  |  | Step 5: 72°C for 10 minutes. |
|  |  | Step 6: 10° forever |
|  |  | Step 7: end |
